# Early grafting in severe adult traumatic brachial plexus injury

**DOI:** 10.3171/2022.10.FOCVID2288

**Published:** 2023-01-01

**Authors:** Justus L. Groen, Willem Pondaag, Martijn J. A. Malessy

**Affiliations:** Leiden NerveCenter, Department of Neurosurgery, Leiden University Medical Center, Leiden, The Netherlands

**Keywords:** brachial plexus injury, early nerve grafting, intraoperative nerve stimulation

## Abstract

Nerve surgical treatment for severe adult traumatic brachial plexus injury is traditionally delayed for months to await spontaneous recovery. Since 2009, the authors have strived to operate on patients with severe brachial plexus lesions within 2 weeks after trauma. This video shows the workup, surgical strategy, and benefits of early supraclavicular nerve grafting, including intraoperative nerve stimulation.

The video can be found here: https://stream.cadmore.media/r10.3171/2022.10.FOCVID2288

## Transcript

In this video we present our treatment strategy, surgical experience, and preliminary results of early supraclavicular grafting in severe traumatic brachial plexus injury. Nerve surgical treatment for adult traumatic brachial plexus injury is traditionally delayed for at least 3 months to await spontaneous recovery. A different, controversial approach is early surgery, which has been advocated by pioneer surgeons such as George Bonney and Rolfe Birch from the UK.^1–4^

### 0:51 Patient Selection.

Since 2009, we adopted a treatment algorithm striving to operate on patients with severe brachial plexus lesions within 2 weeks after trauma.^5^

We perform early surgery on patients suffering from a high-impact crash. This includes patients with C5–8 or C5–T1 lesions, as well as patients with C5–6 or C5–7 lesions with one or more root avulsions on MRI. A prerequisite is that patients are ABC stable and that fractures are treated appropriately.

### 1:24 Imaging.

MRI of the cervical spinal cord is performed as soon as possible after severe brachial plexus trauma. Here you see a T2 MRI series in one of the trauma patients.

The aim of the MRI is to visualize the anterior and posterior root filaments of the affected level.

For reliable information, T2-weighted images on a 3-T machine with a slice thickness of 1 mm are obtained. This MRI clearly shows a pseudomeningocele at the level of C8 and T1 on the right side, with the absence of root filaments.

### 1:46 Positioning and Incision.

The patient is positioned supine, with the ipsilateral leg supported in flexion, to enable the harvest of the sural nerve. Note the typical supraclavicular bruising from the accident. The clavicle is marked and a straight incision 2 to 3 cm cranial and parallel to the clavicle is employed to expose the brachial plexus.

### 2:09 Surgical Exploration.

The surgical field is marked by the sternocleidomastoid muscle medially, the omohyoid muscle caudally, and the trapezius muscle. Also, we can appreciate the external jugular vein and the medial and lateral supraclavicular nerve.

We then work in the medial direction to spot the phrenic nerve as a landmark in the surgical field. The phrenic nerve is stimulated and mobilized and leads to the proximal nerve roots of C4 and C5.

### 2:37 Distal Stumps.

In severe brachial plexus lesions, a variety of lesion patterns can be seen. Here we show some examples of what a supraclavicular exploration may look like in the acute phase.

Case 1: This patient presented with a flail arm on the right side with a Horner sign. At exploration 2 days after trauma, a rupture of the superior trunk (ST) is seen. The distal stump of the superior trunk can easily be dissected and mobilized.

Case 2: In this patient with a left-sided C5–8 lesion, the avulsed nerve roots, typically deflected caudally by the trauma mechanism, can be mobilized. After this, only a small gap between the proximal C5 and C6 stumps and the ruptured superior trunk can be established, facilitating a short graft in reconstruction.

Case 3: In this right-sided complete lesion, one can appreciate the relation of the phrenic nerve and the C5 root: where the phrenic nerve crosses the lateral border of the anterior scalene muscle, the C5 root is found. On the right side, the ruptured ST and avulsed C7 and C8 are seen.

Case 4: In this left-sided complete lesion, the rupture of the upper trunk is more distal, on the level of the divisions. Also, the avulsed roots C7–T1 were pulled to the infraclavicular region. A transpectoral approach facilitates the overview needed for safely mobilizing the retracted plexus parts up to the supraclavicular wound, for reconstruction.

### 4:05 Electrical Stimulation of Distal Nerve.

If surgery is performed in the first 3 days after trauma, one can stimulate the distal nerve to identify the target fascicles. In this case, the stimulation of the C6 root gives biceps contractions and stimulation of C8 leads to finger flexion.

Nerve stimulation in ultra-early surgery even facilitates targeting the desired fascicles in a distal stump: stimulation of the cranial part of C6 leads to contraction of flexor carpi radialis, and stimulation of the caudal side of C6 gives biceps contraction.

### 4:46 Preparation of Distal Stump.

After dissection and visual check of the fascicular structure of the distal nerve, stimulation can prove the patency of the distal stump.

### 4:55 Preparation of Proximal Stump.

The proximal stumps are then cut until clear intraneural anatomy of the fascicles is visible, using a No. 10 scalpel blade.

### 5:05 Harvest of Sural Nerve Graft.

The ipsilateral sural nerve is taken out using three to five short incisions. The leg is supported in flexion. The sural nerve is identified distally midway between the lateral malleolus and the Achilles tendon, and then neurolyzed from the subcutaneous layer toward its proximal origin. Adhesions and small branches are cut. The nerve is identified in the next incision by gentle traction using vessel loops. The branching pattern and attributions from peroneal and tibial nerve varies. We attempt to harvest as many branches as possible for maximal graft length.

### 5:45 Supraclavicular Grafting.

The most common pattern in complete lesions is avulsions of lower plexus elements with rupture of C5 and C6. In this situation, preferably C5 is used as proximal stump for grafts to the suprascapular nerve and PD-ST, and C6 to AD-ST.

The lesion pattern determines the reconstruction strategy. In this next case, C8 function was spared, and the only viable stump of C5 was therefore used for neurotization of exorotation and abduction function.

In this case, a transfer from C4 to the postganglionic fibers of C7 is performed, to gain some hand sensation. These transfers are only feasible in early surgery when ganglion anatomy can still be appreciated.

In this case, with a more distal rupture of the superior trunk, an effort is made to reanimate hand function. Therefore, we made a direct coaptation of the anterior division of the superior trunk to the avulsed C8 nerve root.

### 6:53 Rationale Behind Early Grafting.

Nerve surgery for adult brachial plexus lesions is often delayed for 3 to 4 months. But why wait when you know there is a rupture or root avulsion?

Early surgery has clear surgical advantages. Scarring has not occurred yet, which facilitates dissection, especially when surgery is performed within 1 week after trauma. Early surgery allows better mobilization of distal stumps, which results in a smaller nerve gap and thus shorter grafts. Direct electrical stimulation of distal stumps is possible when surgery is performed within 3 days, which will aid in identification of distal stumps and allows improved distal targeting. Due to the shorter interval, less degeneration will have occurred in the proximal nerve, the distal nerve, and in the target muscle. Additionally, an earlier diagnosis results in less uncertainty for the patient, enabling a better rehabilitation strategy.

Eventually, a proper evaluation of results should show superior results of early surgery compared to waiting attitude.

Early surgical treatment has been performed in our clinic since 2009.^5^ We managed to operate on around one-quarter of patients within 2 weeks after injury; half of our patients were surgically treated within 3 months. Late surgeries were usually due to referral delay.

### 8:11 Results in Complete BPI Lesions.

We performed a preliminary analysis of our results. We analyzed patients with C5–T1 lesions, in whom we performed a nerve grafting procedure. The primary outcome measure is MRC grade of the biceps muscle after 2 years.

A good result was defined as MRC grade 4, which was more often reached in patients who were surgically treated early. When we analyzed fair outcomes, defined as MRC 3 or better, there was less difference comparing the ultra-early group with later surgery.

A clear difference was a smaller graft length needed to bridge the defect in patients that were operated on within 2 weeks.

### 8:50 Challenges of Ultra-Early Surgery.

Ultra-early surgery brings new challenges to surgical expertise. As it concerns multitrauma patients, a prerequisite is the patient is ABC stable and that fractures have been treated adequately.

Posttraumatic swelling may still be present, resulting in diffuse oozing from the surgical field. The extent of the nerve lesion needs to be judged differently in the acute setting compared to the chronic phase. As surgeons, we must learn a new appreciation of demarcation in proximal and distal stumps.

Direct electrical stimulation of the distal stump may be helpful.

Special attention is needed to check for CSF leakage through the foramen of avulsed roots, in which case the foramen must plug carefully using fat or muscle, and afterward sealed with fibrin glue.

### 9:41 Summary.

In summary, early nerve surgery brings more opportunities than difficulties. Our preliminary results are promising, so we would encourage our viewers to adopt this early surgical approach.

## Acknowledgments

We thank Ms. Wynn Heliczer for the voice-over of this video.

## Disclosures

The authors report no conflict of interest concerning the materials or methods used in this study or the findings specified in this publication.

## Author Contributions

Primary surgeon: all authors. Assistant surgeon: Malessy. Editing and drafting the video and abstract: Pondaag, Groen. Critically revising the work: Malessy. Reviewed submitted version of the work: Pondaag. Approved the final version of the work on behalf of all authors: Pondaag. Supervision: Malessy.
